# A symphony of inner ear developmental control genes

**DOI:** 10.1186/1471-2156-11-68

**Published:** 2010-07-16

**Authors:** Sumantra Chatterjee, Petra Kraus, Thomas Lufkin

**Affiliations:** 1 Stem Cell and Developmental Biology, Genome Institute of Singapore, 60 Biopolis Street, 138672, Singapore; 2 Department of Biological Sciences, National University of Singapore, 117543, Singapore

## Abstract

The inner ear is one of the most complex and detailed organs in the vertebrate body and provides us with the priceless ability to hear and perceive linear and angular acceleration (hence maintain balance). The development and morphogenesis of the inner ear from an ectodermal thickening into distinct auditory and vestibular components depends upon precise temporally and spatially coordinated gene expression patterns and well orchestrated signaling cascades within the otic vesicle and upon cellular movements and interactions with surrounding tissues. Gene loss of function analysis in mice has identified homeobox genes along with other transcription and secreted factors as crucial regulators of inner ear morphogenesis and development. While otic induction seems dependent upon fibroblast growth factors, morphogenesis of the otic vesicle into the distinct vestibular and auditory components appears to be clearly dependent upon the activities of a number of homeobox transcription factors. The *Pax2 *paired-homeobox gene is crucial for the specification of the ventral otic vesicle derived auditory structures and the *Dlx5 *and *Dlx6 *homeobox genes play a major role in specification of the dorsally derived vestibular structures. Some Micro RNAs have also been recently identified which play a crucial role in the inner ear formation.

## Review

### Introduction

**Figure 1:**
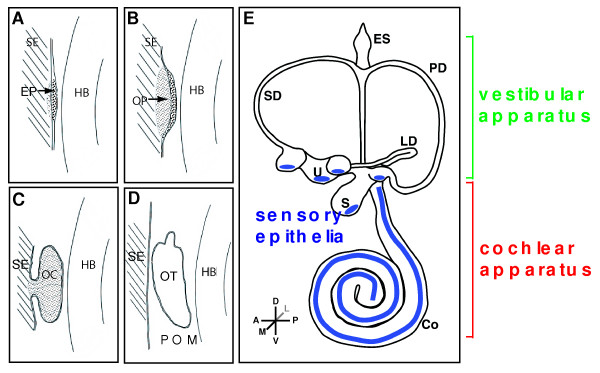
**Developmental milestones in mouse inner ear formation**. Competence of surface ectoderm lateral to both sides of the hindbrain (HB) precedes any cell morphology changes. (A) Thickening of surface ectoderm (SE) to form the early placodes (EP) which is primarily driven by *Fgf, Wnt *and *Pax *genes. (B) Invagination of the otic placodes to form the otic pit (OP). (C) Further development and invagination of the otic pit to form the otic cup (OC) which pinches off from the surface ectoderm. (D) The separation from the overlying ectoderm gives rise to the otocyst (OT). (E) Subsequent morphogenesis to finalize the complex 3-dimensional labyrinth which is demarcated into vestibular and cochlear components. Sensory epithelia are shown in blue. Abbreviations: Co, cochlea; ES, endolymphatic sac; HB, hindbrain; LD, lateral semicircular duct; PD, posterior semicircular duct; POM, periotic mesenchyme; S, saccule; SD, superior semicircular duct; SE, surface ectoderm; U, utricle.

**Figure 2:**
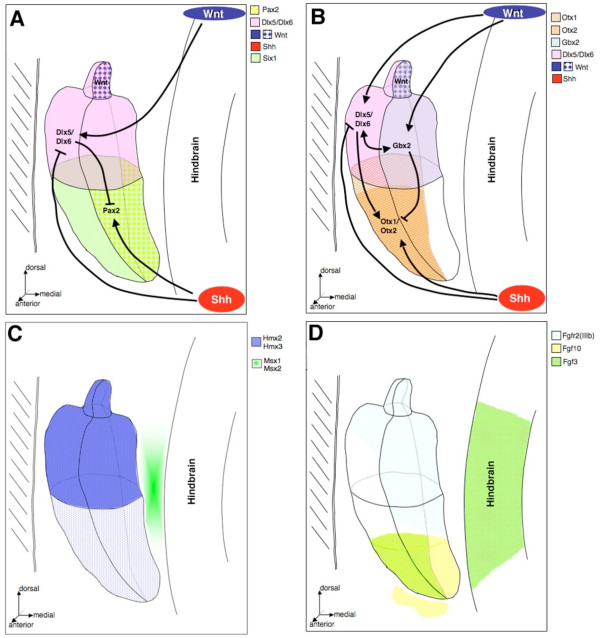
**Representative expression patterns of genes controlling cochlear and vestibular specification**. (A) Shh functions to maintain *Pax2 *and restrict *Dlx5/Dlx6 *in the medial wall of the otic vesicle in order to specify cochlear fate. *Dlx5/Dlx6 *specify the medial to dorsal most cells of the otic epithelium that give rise to the endolymphatic duct and vestibular apparatus. (B) Secretion of Shh from the notochord specifies the ventral most cells of the otic epithelium that express *Otx1/Otx2 *and possibly *Pax2 *which contribute to cochlear morphogenesis and outgrowth. In addition, *Dlx5/Dlx6*-dependent vestibular specifications and morphogenesis is dependent upon the activation of *Gbx2 *and *Bmp4 *function (not shown) and partial activation/expression of *Otx1*. *Dlx5/Dlx6 *also functions to restrict *Pax2 *expression to the medial wall of the otic vesicle epithelium. Thus, *Dlx5/Dlx6 *and *Shh *may functionally antagonize each other, through repression, to generate compartments of activities that specify the vestibular and cochlear cell fates. (C) Both *Hmx2 *and *Hmx3 *are required for cell fate determination and subsequent morphogenesis of the developing inner ear. Loss of both *Hmx2 *and *Hmx3 *results in the absence of the entire vestibular system. *Msx1/Msx2 *are expressed in the adjacent periotic mesenchyme and are critical for middle ear development. (D) *Fgfs *function with *Shh *in the periotic mesenchyme to initiate ventral otic capsule chondrogenesis via *Brn4 *and *Tbx1 *function (not shown). *Fgfs *are also expressed in the hindbrain epithelium adjacent to the otocyst and are important for induction of the otic placode.

**Figure 3:**
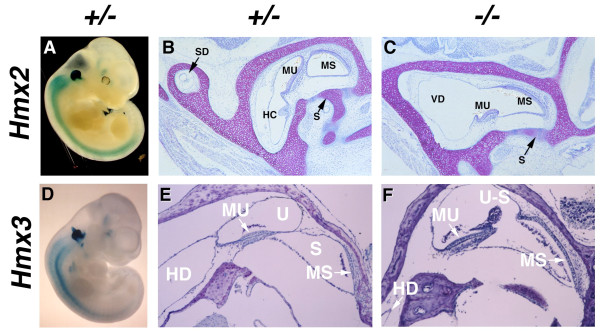
**Abnormal vestibular structure and morphogenesis in whole-mount β-galactosidase stained mid-gestation embryos lacking either *Hmx2 *or *Hmx3***. Early in development *Hmx2 *(A) is expressed throughout both the vestibular portions of the inner ear of heterozygous (A) embryos. Embryos that are homozygous for the absence of *Hmx2 *(C) have relatively normal cochlear development in the presence of severely dysmorphic vestibular development. The endolymphatic duct morphogenesis is retarded and the superior (SD), posterior (PD), and lateral or horizontal (HD) semicircular ducts appear to form a fused and primitive vestibular diverticulum (VD) and is associated with decreased maculae of the utricle (MU) and saccule (MS). In contrast, *Hmx3 *expression in the inner ear of heterozygous (D) and homozygous (F) embryos demonstrates expression throughout only the vestibular apparatus, including the ED and all three semicircular ducts. Embryos that are homozygous for the absence of *Hmx3 *(F) have mild faulty development of vestibular structures including a fusion of the utricular and saccular chambers (U-S) and a dysmorphic utricular maccula (MU) in the presence of circling behavior.

Imagine yourself at a symphony concert in the midst of an exited audience, alone in permanent silence; silence resulting from an inner ear defect. Or consider the feeling after a whirling rollercoaster ride when your senses are left "off balance". The mammalian inner ear is a complex structure functionally organized into auditory and vestibular components that are responsible for detecting and coordinating the senses of hearing, acceleration and balance. The mature mammalian inner ear has two major components, the vestibular and auditory organs. The vestibular organ senses balance and changes in movement. It contains the three semicircular canals that sense angular acceleration and the utricle and saccule, both of which are responsible for sensing gravity and linear acceleration. The auditory organ consists of the coiled cochlea, which senses sound. Within both of these organs a specialized sensory epithelium converts mechanical actions into electrical potentials. These epithelia contain sensory hair cells (HC) -mechanoreceptors that initiate action potentials in response to sound or movement- as well as surrounding supporting cells. Damage to this small population of hair cells is a major cause of hearing loss. There are numerous other cell types in the inner ear that are also required for the mechanical, electrical, and structural aspects of hearing and balance. Examples of such cell types are the nonsensory supporting cells surrounding the hair cells [1], those of the stria vascularis on the lateral wall of the cochlear duct, responsible for the production of the endocochlear electrical potential [2], and those of the various membranes on which the sensory organs rest and that separate the different compartments of the inner ear. Over the years several gene mutations have been identified resulting in deafness, impaired hearing or vestibular dysfunction [3,4]. A better understanding of inner ear development and its associated genomics and proteomics will facilitate a better understanding of the many causes of deafness and vertigo. Development of the inner ear follows a theme common also to many other anlagen of forming appendages (e.g. lens, teeth and hair, Figure 1): (1) Ectodermal-/mesenchymal cross talks lead to the **initiation of a placode **(Figure 1A); (2) **Invagination **of the placode to form the otic cup or pit (Figure 1B), and in mice and chick complete separation from the surface ectoderm to form a drop-shaped otic vesicle or otocyst (Figure 1C,D); (3) **Patterning and differentiation **of the otocyst (Figure 1E). During all steps of inner ear formation we reencounter well known critical regulators of vertebrate development, many of them homeobox genes which carryout other roles in different tissues of the organism. Interaction between all these players has to be perfectly orchestrated along the three major body axes (anteroposterior, dorsoventral and mediolateral) to allow the formation of a structure as complex and rich in detail as the inner ear. Hence "listening" to the symphony of developmental control genes during inner ear development will contribute to our understanding of the complex interaction of these key performers in embryonic development in general.

### The rhythm of the different genes

Like any philharmonic orchestra with many musicians the development of the inner ear is a concerted effort of many genes working in harmony to create a perfectly balanced organ. One of the major groups of genes which play a key role in the development of the inner ear is the homeobox gene family, characterized by their 180 bp homeodomain.

During vertebrate inner ear formation members of the ***Pax *paired-homeobox gene **family (Figure 2A), mammalian homologs to the *Drosophila Paired *(*Prd*) gene play a very crucial role. In vertebrates, there are nine known *Pax *(*Pax1-Pax9*) genes that can be subdivided into groups according to conservation of the paired box sequence [5]. Mammalian ***Otx *homeobox genes **(Figure 2B) homologs of the *Drosophila Orthodenticle *(*Otd*) gene that have shared developmental roles crucial for specification and regionalization of the forebrain and midbrain [6] also play a critical role in inner ear development. Members of the ***Gastrulation brain homeobox *(*Gbx*) **family (Figure 2B), a mammalian homolog of the *Drosophila Unplugged *(*Unp*) gene and the ***Msx *homeobox gene **family members are homologs of the *Drosophila Muscle segment *gene are also important for proper development of the inner ear. The three mammalian *Msx *genes have overlapping expression patterns and possess diverse functions during embryogenesis by primarily acting as transcriptional repressors through interactions with transcriptional complexes or with other homeodomain proteins (Figure 2C) [7,8]. There are six known ***Six *homeobox genes **(*Six1-Six6*), they are mammalian homologs to the *Drosophila Sine oculis *(*So*) gene, which participate in an evolutionarily conserved gene network consisting of *Pax*-*Eye absent *(*Eya*)-*Six*-*Dachshund *(*Dach*) gene family members and are expressed during the development of numerous organ systems (Figure 2A) [9]. They interact with the *Eya *family of proteins via protein-protein interactions across a wide range of species during organogenesis of a multitude of tissues [10-12]. ***Hmx *homeobox genes **were first identified in humans and have homologs in a number of species including *Drosophila *[13,14], zebrafish and medaka[15-17]. There are three known mammalian *Hmx *genes (*Hmx1-Hmx3*) that are also related to the chick *Sensory organ homeobox-1 *(*SOHo-1*) gene, based on amino acid homology, and are expressed throughout the developing central and peripheral nervous systems (Figure 2C) (reviewed in [13,18,19]. And last but not least, the ***Dlx *homeobox genes **are homologs of the *Drosophila Distal-less *(*Dll*) gene and encode transcription factors that appear to have critical developmental functions in all species and tissues, in which they are expressed (Figure 2A) [20,21]. In *Drosophila*, *Dll *is a critical upstream regulator of sensory and non-sensory development of appendages and antennae, as loss of *Dll *or its downstream target genes results in antennae with sensorineural hearing loss [22]. The six identified mammalian *Dlx *genes are convergently transcribed gene pairs (*Dlx1/Dlx2*, *Dlx5/Dlx6*, and *Dlx3/Dlx7*) that have overlapping regulatory elements and expression patterns. In humans, the paired *Dlx5/Dlx6 *genes, which map to chromosome *7q22*, are postulated as candidates for split hand/split foot malformation (SHFM1) [23-26]. Furthermore, sensorineural deafness and vestibular malformations are also associated with SHFM1 [27-30].

Signaling during inner ear development requires more than the homeobox gene families. No signaling network would be complete without members of the ***Tgfbeta ***super family, the ***Fibroblast growth factor (Fgf) ***family (Figure 2D), ***Sonic hedgehog (Shh)***and its target genes as well as the ***Wnt***and ***Notch ***signaling cascades. Further crucial players are members of the ***Forkhead (Fox) *genes, **a family of winged-helix transcription factors and ***T-box (Tbx) *genes**, a family of mouse *Brachyury *and *Drosophila Optomotor-blind *(*omb*) homologs that encode transcription factors that contain a conserved 180 amino acid T-box DNA binding domain [31,32]. More than 40 T-box genes have been identified that have evolutionary conserved functions during embryonic development in a wide range of animals.

### Many are on stage to form an otic placode, but few get to play

The mammalian inner ear is a complex structure. Formation of the inner ear becomes apparent in mouse around E7.5/E8, when cranial ectodermal thickenings, the otic placodes, are symmetrically located on opposing sides of the hindbrain. Induction of the placodes is the outcome of cross talk mediated by signaling events originating from the presumptive otic epithelium and underlying periotic mesenchyme, the adjacent notochord and neural tube in the region spanning rhombomeres four to six [33,34].

There is substantial evidence that *Fgf *signaling plays a critical role in the induction of otic tissue in vertebrates, however, despite *Fgf3 *being a "hot" candidate [33] labeling a single *Fgf *as ***the ***otic inducer has proven difficult since functional redundancy already observed for *Fgf *family members in other developmental anlagen (e.g. limb) is also apparent in the ear: *Fgf3 *loss-of-function does not appear to affect ear development, while *Fgf10 *mutations do result in inner ear defects. *Fgf10 *null mutants show complete agenesis of the posterior canal crista and the posterior canal. The posterior canal sensory neurons form initially and project rather normally by E11.5, but they disappear within 2 days. *Fgf10 *null mutants have no posterior canal system at E18.5. In addition, these mutants have deformations of the anterior and horizontal cristae, reduced formation of the anterior and horizontal canals, as well as altered position of the remaining sensory epithelia with respect to the utricle [35]. Combined *Fgf3/Fgf10 *mutants do not form an otocyst and show severe impact on the expression of *Pax, Dlx *and *Otx *family members. New findings suggest that quantitative gene dosage of combined *Fgf3 *and *Fgf10 *signaling are essential for otic placode induction in mouse (Figure 2D) [36,37]. Interestingly, otic placode induction appears to result from an interplay between *Wnt *and *Fgf *signaling, reminiscent of what has been observed during early limb development [33,38,39]. In contrast to the short-range interaction in flies, Shh and Wnts are expressed in the floor plate and roof plate, respectively, of the developing brainstem and spinal cord of vertebrates [40-42]. *Wnt *genes are co-expressed with *Bmp4 *and *Gli3*. Combined, *Bmp4*, *Gli3 *and *Wnt *genes antagonize the ventralizing effects of SHH and give a dorsal identity. This countergradient set up by these genes allows for functional roles of various downstream genes for regional identity. Given the proximity of the ear to the hindbrain, the patterning genes in the hindbrain will affect the ear development as shown in some classical experiments [43]. For a detailed review see [44].

*Pax2 *and *Pax8 *are some of the earliest known genes to be expressed in the pre-otic tissue [45,46]. Its onset of expression suggested *Pax8 *as a critical regulator of otic induction and subsequent morphogenesis. However, *Pax8 *null mice have normal inner ear development [47]. There is the possibility that *Pax8 *function during otic development is masked by redundant function of an unrelated gene or by another member of the *Pax *gene family. Initially broad preplacodal *Pax2 *expression becomes ventrally restricted medial epithelial expression in the developing otocyst (Figure 2A), while *Pax8 *expression is maintained throughout the formation of the otic vesicle [48,49].

While *Fgf, Wnt *and *Pax *genes have been implicated in otic induction, other gene families are also expressed during that early stage, however no obvious genetic function could be assigned to them. This could be either due to functional redundancy or because their critical role does not unfold until much later, when otic development is well on its way. In chick, *Msx1 *expression marks the medial edge of the preotic placodal region adjacent to the hindbrain and prior to the onset of *Pax2 *expression [50]. Only a subset of the *Msx1 *expressing cell population actually contributes to the formation of the otic placode and the role of *Msx1 *expression during otic induction and morphogenesis remains unreported. However it is not unusual to find *Dlx *and *Msx *genes expressed in close proximity to one another, as they are often thought to function together in gene regulation [51]. *Foxi1 *could play a role in vertebrate otic placode formation given that in *foxi1 *zebrafish mutants morphology of the otic placodes as well as the expression of *pax2/8 *and *dlx3b/4b *is affected [52]. However, in mice otic placode development appears normal in *Foxi1 *loss of function mutants [53]. While its role might be over shadowed by functional redundancy with *Foxf2*, which is also expressed early in the otic epithelium in mouse [54], a critical function during early otic development in mouse remains questionable. Studies have shown that *Foxg1 *is expressed in most cell types of the inner ear of the adult mouse and that Foxg1 mutants have both morphological and histological defects in the inner ear[55]. These mice have a shortened cochlea with multiple rows of hair cells and supporting cells. Additionally, they demonstrate striking abnormalities in cochlear and vestibular innervation, including loss of all crista neurons and numerous fibers that overshoot the organ of corti. Recent studies have also shown the critical role of *Foxg1 *in sensory cristae[56]. Genetic fate-mapping analyses indicate an improper separation between anterior and lateral cristae in *Foxg1 *^**-/- **^mouse mutants. The data suggest that a function of *Foxg1 *in the inner ear is to restrict sensory fate which is in conflict with previous data proposing that sensory cristae induce formation of their non-sensory components, the semicircular canals. *Hmx2 *and *Hmx3 *(also known as *Nkx5.2 *and *Nkx5.1*, respectively) are coexpressed in the otic placode, with *Hmx3 *being expressed in the otic epithelium starting at E8.5, just a few hours prior to the onset of *Hmx2 *[57]. At the same time, *Gbx2 *expression becomes detectable in the otic placode [37,58]. Of the *Dlx *gene family, only *Dlx5/Dlx6 *are expressed in the pre-placodal and throughout the otic placode stage [59,60]. So are *Six1 *and *Six4*, members of the *Six *homeobox gene family, however their main function seems to be exerted at subsequent stages of otic development (for review see [33].

### From placode to otocyst

Induction of the otic placode is followed by its invagination to form the so called otic cup or otic pit, which in mouse encloses and separates from the surface ectoderm to create a drop shaped otic vesicle or otocyst by E9 (Figure 1). Otic patterning has to be precisely coordinated along three axes: Anteroposterior (AP), dorsoventral (DV) and mediolateral (ML). Data generated in recent years largely supports a 'compartment-boundary' model of cell fate specification and patterning in the inner ear [61], suggesting that once the otic vesicle forms, regions have been established and the otic epithelium is compartmentalized along all three axes as indicated by restricted expression profiles of genes like *Pax2, Fgf3, Lunatic fringe, Six1, Bmp4 *and *Bmp *antagonists [61-64]. The inter compartment boundaries can mediate local patterning and cell fate decisions in the otocyst [61]. This is supported by observations that distinct regions identified by their gene expression patterns in the developing otocyst give rise to a particular component of the inner ear. Subsequent loss of gene function studies in mouse have shown varying genetic roles in establishing inner ear structures predicted by the compartments [61,64]. Most importantly, these studies have shown a vast role for numerous homeobox genes in establishing compartmental boundaries, subsequent to otic induction, and in specifying cell fates during morphogenesis of the inner ear from compartmentalized domains of gene expression [57,65-69].

As members of the *Pax-Six-Eya-Dach *signaling network and considering the early onset of expression, *Six1 *and *Six4 *are likely to contribute to compartmentalization and specification of ventrally derived auditory structures following induction of the otic placode. *Six1 *and *Six4 *continue to be expressed during the otic vesicle stages of inner ear development. Insight into their functional role during mammalian inner ear development was obtained through the analysis of *Eya1 *null mice, as *Six1 *expression is concomitantly lost during the total regression of inner ear structures [12,70]. *Six4 *has also been demonstrated to interact with *Eya1 *in the mouse [71]. However, its functional role remains unclear since *Six4 *null mice have normal inner ear development, which may be due to functional compensation by *Six1 *[72]. *Six1 *expression is restricted to the ventral half of the otic vesicle that gives rise to the auditory components of the inner ear (Figure 2A). Recent analysis of two independent *Six1 *null mouse strains indicates an important role for establishing and/or maintaining compartmental boundaries and in cochlear specification [73,74]. While otic vesicle formation occurs, development beyond this stage is affected in the *Six1 *null mice. On the level of gene expression a dorsalization of the ventral otocyst is observed: ventral markers such as *Fgf3, Fgf10*, *Otx1, Otx2 *and *Lunatic Fringe *are lost and the dorsal markers *Hmx3 *and *Dlx5 *are expanded ventrally (Figure 2, for review see [75].

As early as E10.25, *Otx1 *and *Otx2 *are expressed in the posteroventrolateral and ventral apex, respectively, of the otic epithelium following formation of the otic vesicle [61,76]. The ventral apical domain serves as an area of overlapping expression. The ventral cells of the otic epithelium are believed to be fated to give rise to the cochlear duct and organ of Corti [12]. *Otx1 *null mice have cochlear and saccular defects that are consistent with its ventral expression domain [77,78]. Furthermore, the defects expand dorsally to the lateral semicircular duct and its ampulla that would also be predicted by a 'compartment-boundary' model of cell fate specification [77,79]. Notably, *Otx1/Otx2 *expression is abutting areas positive for the characteristic sensory markers *Bmp4 *and *Fng *[77]. At the other pole, *Gbx2 *is expressed in the dorsomedial otocyst. In the absence of *Gbx2*, *Wnt2b*, a marker of the developing endolymphatic duct, is lost. Furthermore *Gbx2 *appears to be required to maintain *Dlx5 *expression regionally, since *Dlx5 *is absent from the medial but not lateral otocyst (Figure 2B) [80]. Its compartment of otic vesicle expression is predicted to have a role in development of the endolymphatic duct and in establishing the dorsal boundary of the saccule sensory compartment [81]. Interestingly, unlike in mid-hindbrain patterning, where *Otx2 *and *Gbx2 *expression abuts one another, with this juxtaposition being critical for the positioning of the organizer, in the otocyst, *Otx2 *and *Gbx2 *sandwich the presumptive sensory patches and *Lunatic fringe (Lfng) *expression [61,80].

By E9.5 in mouse *Hmx2 *and *Hmx3 *expression becomes compartmentalized to the dorsolateral epithelium of the otic vesicle (Figure 2C) [57,69]. In chick, lateral expression of *SOHo-1 *in the otic vesicle marks the epithelial territories that give rise to the presumptive semicircular ducts and their cristae [61,82,83]. In zebrafish the onset of *Hmx3 *expression in the otic vesicle and lateral line organs starts at 11.5 hpf and *Hmx2 *expression in the same tissue is detected at 14 hpf [17]. Similarly, upon formation of the otic vesicle *Dlx5/Dlx6 *expression becomes restricted to the dorsal hemisphere (Figure 2A,B) [26,60,65,66]. The dorsomedial expression domain of *Dlx5 *and presumably *Dlx6 *are restricted by the function of *Shh*, which is secreted onto the otic epithelium from the nearby notochord [84]. The cells compartmentalized within the otic vesicular *Dlx5/Dlx6 *expression domain are fated to give rise to the vestibular apparatus, according to the 'compartment-boundary' model of inner ear development [63].

The T-box gene *Tbx1 *is initially expressed in the otic vesicle epithelium and subsequently in the periotic mesenchyme. The mechanistic role of *Tbx1 *remains unclear, but its expression appears to be critical for morphogenesis, as the inner ears of *Tbx1 *null mice have the morphology of an undifferentiated otic vesicle with normal endolymphatic duct formation [85]. With neuronal precursors originating from the anterior otocyst, more recent *Tbx1 *gain and loss of function studies further suggest a role in regulation of neurogenesis via regulating anteroposterior axis development in the otocyst [86].

### Shaping of the inner ear into auditory and vestibular structures

The mature inner ear with its elaborately designed acoustic and vestibular apparatus is encased in the dense bone of the skull. Molecular and fate mapping data created in recent years shines some light into this cave and helps to understand the formation of such complex structures evolving from a "simple" drop-shaped otocyst.

While the otocyst initially consists of simple pseudo-stratified epithelium it soon undergoes extensive proliferation, differentiation and morphogenesis that will eventually establish the ventrally derived auditory component, the cochlea, and the dorsally derived vestibular apparatus. In mammals, auditory perception is initially mediated through sensory cells located in a rigorously patterned mosaic of unique cell types located within the coiled cochlea. Almost all of the cell types within the membranous labyrinth of the inner ear are derived from multipotent epithelial progenitor cells initially located in the otocyst. Otocyst-derived cells develop into three major lineages, prosensory (cells that will develop as either hair cells or associated supporting cells), proneural (cells that will develop as auditory or vestibular neurons) and nonsensory (all other otocyst derived cells) with cells within each lineage developing in different spatio-temporally defined domains of the otocyst. Recent results have identified specific signaling molecules and pathways, including *Notch*, *Hedgehog*, *Sox2 *and *Fgfs*, that guide progenitor cells to develop first as a sensory precursor and subsequently as one of the more specialized cell types. For a detailed review on cochlear development see [87,88]. Highly differentiated sensory hair cells develop within the coiled cochlear duct to form the organ of Corti, which is responsible for detecting sound. Likewise, sensory hair cells arise within the vestibular apparatus to form the maculae in the utricle and saccule and the cristae in the semicircular ducts (Figure 1E). Collectively, they are responsible for detecting gravity as well as linear and angular acceleration, which all function coordinately to maintain balance [12,89] for review see [90]. An organ so complex in function and structure and at the same time so rich in detail as the mature inner ear requires absolute precise coordination of all the developmental genes and signaling cascades involved. Generally and as predicted from the "compartment boundary model" genes expressed in the ventral otocyst will be implicated in formation of auditory structures, while genes expressed in the dorsal otocyst are implicated in the formation of the vestibular apparatus.

*Pax2 *which we got to know as an early marker of otic fate, is subsequently required for cochlear development, and its inactivation in mice leads to cochlear agenesis [68]. *Pax2 *is also expressed in the endolymphatic duct [91]. However, the vestibular apparatus and endolymphatic duct develop normally in the *Pax2 *null mice, maybe due to redundancy with other *Pax *genes, possibly *Pax8*. The future generation and analysis of *Pax2/Pax8*-null mice could substantiate and clarify the roles of both genes during inner ear development.

In the *Otx1 *null mice, both the lateral semicircular duct and the lateral sensory cristae are absent, similar to *Prx1/Prx2 *double-null mice suggesting that *Otx *and *Prx *genes may interact with each other via unknown secretable factors during inner ear development. The ventral-apical expression domain of *Otx2 *in the otic epithelium gives rise to the saccule and a portion of the cochlea. Since *Otx2 *null mice die prior to morphogenesis of the inner ear beyond the otic vesicle, *Otx2 *function during inner ear development has been inferred by the analysis of *Otx1 *null mice that are also heterozygous for *Otx2 *[77,92,93]. The inner ear defects in these mice are progressively more severe than those reported for the *Otx1 *null mice [77]. The defects to the cochlea are expanded ventrally and the saccule, which is unaffected in *Otx1 *null mice, is dysmorphic as might be predicted by the 'compartment-boundary' model. Therefore *Otx2 *expression apparently functions both redundantly and independently of *Otx1 *in establishing proper specification of the cochlea and saccule. Unlike *Otx1*, *Otx2 *expression does not appear to be mediated by Shh signaling as ectopic expression of *Shh *in mice does not induce concomitant ectopic expression of *Otx2 *[84]. A more detailed analysis of the involvement of *Otx2 *expression during inner ear specification and patterning may require the generation of conditional loss of function mutants or alternative systems.

*Gbx2 *expression eventually becomes restricted to the endolymphatic duct and ceases in the inner ear by E15.5 [80]. The loss of function analysis confirmed phenotype predictions based on the compartment boundary model and showed a key role of *Gbx2 *in patterning dorsomedial (endolymphatic duct, vertical pouch) [80] leaving *Gbx2 *null mice with a phenotype similar to that described for *kreisler *mice ([94] and references therein) namely absence of the endolymphatic duct and swelling of the membranous labyrinth. In more severe cases ventral inner ear structures (saccule, cochlea) were also affected [80].

As predicted by its expression domain, *Hmx3 *is required for proper specification of structures within the vestibular apparatus. In *Hmx3 *null mice, vestibular defects include the anterior and posterior semicircular ducts being severely reduced or lost and the lateral semicircular duct always being absent, while auditory development is unaffected and functional [95]. These mice display features of hyperactivity and circling that phenocopy the *shaker/waltzer *mutant mice (Figure 3). In contrast, a second *Hmx3 *null mouse strain has relatively normal semicircular duct formation in the presence of a similar circling behavior [69]. These mice have utricles and saccules that are fused into one chamber that has a severe reduction and/or absence of sensory epithelial cells within their maculae. In addition, their entire lateral semicircular duct cristae are completely absent. The variable phenotypes suggest that other factors may have the ability to functionally compensate for *Hmx3 *in a dose-dependent manner during vestibular specification (Figure 3). Loss of *Hmx2 *gene function in mice demonstrated a pronounced role in vestibular development as 65% of *Hmx2 *null mice display hyperactivity, head tilting and circling behavior in the apparent absence of central nervous system defects [57]. Histological and molecular analysis reveals inner ear defects that are significantly more severe than those observed in both *Hmx3 *null mouse strains. Morphogenesis of the pars superior portion of the otic vesicles arrests following the formation of the primordial vestibular diverticula, which results in the complete absence of all semicircular ducts, fusion of the utricles and saccules, and significant loss of the vestibular sensory epithelium (Figure 3). The vestibular defects appear to arise from deficient proliferation within the otic epithelium and periotic mesenchyme that leads to abnormal *Dlx5 *and *Bmp4 *expression patterns. In addition, the expression domain of *Pax2 *is reduced, but cochlear development proceeds normally in the *Hmx2 *null mice. Since the available data indicates that *Dlx5*, *Bmp4 *and *Pax2 *are unlikely downstream targets, *Hmx2 *appears to be required for maintaining the commitment of a subpopulation of otic epithelial cells that specify the entire vestibular apparatus. Taken together, *Hmx2 *is likely to be able to functionally compensate for *Hmx3 *during semicircular duct formation, but not completely during morphogenesis of the maculae and cristae. Thus, *Hmx2 *and *Hmx3 *have unique and redundant functions in the specification of cells that generate a set of sensory and non-sensory vestibular structures.

Double knockdown studies of *Hmx2/Hmx3 *in zebrafish have reported the appearance of fused otoliths and the loss of lateral line neuromasts (at 3 dpf) and rescue experiments with capped RNA have demonstrated the redundancy of these two genes in formation of lateral neuromast[17].

Loss of function mutations in *Dlx1*, *Dlx2*, or *Dlx1/Dlx2*and *Dlx7 *have not been reported to cause any defects in inner ear development and can not be studied in *Dlx3 *due to early embryonic lethality [59,96]. However, functional loss of the *Dlx5 *gene directly affects the morphogenesis of sensory and non-sensory vestibular structures and is postulated to indirectly affect distal cochlear development [65,66]. Variable vestibular defects range from the absence of one to all three semicircular ducts, impaired cristae formation, and a consistent shortening of the endolymphatic duct, while the utricle and saccule develop with slightly abnormal maculae. The vestibular defects seem to arise from deregulation of pathways controlling temporal and spatial patterns of both cellular proliferation and apoptosis that govern differentiation and specification of the inner ear. Furthermore, these mechanisms appear to be controlled by epithelial-mesenchymal interactions.

Mammalian *Msx1/Msx2 *are expressed at diverse sites of epithelial-mesenchymal interactions, including within the otic epithelium and periotic mesenchyme [60,97,98]. *Msx1 *null and *Msx1/Msx2 *double-null mice have middle ear defects with a respective increase in severity and apparently have functional redundancy as *Msx2 *null mice do not have middle ear defects [97,99,100]. Surprisingly, inner ear and otic capsule defects have not been reported for any combination of *Msx1/Msx2 *double-null mice even though the double-null mice have severe craniofacial defects [99]. There is intriguing evidence in chick that *Msx1 *expression in the otic epithelium may contribute to both sensory and non-sensory vestibular development [101]. Additional experimentation is required to determine if any combination of *Msx1/Msx2 *regulates or modulates other factors during inner ear morphogenesis and/or otic capsule formation.

Analyses of the zebrafish *van gogh *(*vgo*) mutation and mouse models of the human DiGeorge (velo-cardio-facial) syndrome, which includes conductive and sensorineural hearing loss, have revealed that disruptions in *Tbx1 *expression have detrimental effects on outer, middle and inner ear development [102-104]. In addition to aberrant otic vesicle formation, there are lost expression domains of *Pax2, Otx2, Fgf10 *and *Bmp4 *within the otic epithelium. However, rather than being downstream target genes of *Tbx1 *it appears that their abnormal expression is the result of lost cell population(s). Furthermore, *Tbx1 *expression in the periotic mesenchyme, but not in the otic epithelium, is lost in *Shh *null mice in a manner that strongly implies a functional role in otic capsule formation [84]. Additional investigations are required to determine if the functions of *Tbx1 *in the epithelium and mesenchyme are linked and if they are required for the expansion/differentiation of a subpopulation of otic epithelia cells that specify the cochlea and vestibular apparatus.

### "Sense" the rhythm of the inner ear

Sensory cells in vertebrates either have an axon or are innervated by placode derived neurons associated with "secondary" sensory cells such as in the inner ear, lateral line etc. Concurrent with the role of *Fgf10*, development of inner ear neurons depends critically on the bHLH genes *Neurog1 *[105] and *Neurod1 *[106]. *Neurog1 *and *Neurod1 *are also necessary for olfactory receptor development in mice [107] thus indicating that *Neurog1*, while necessary for ear placode derived neuron formation, is not sufficient to identify such neurons. Placodally derived sensory neurons use the Pou domain factor *Pou4f1 (Brn3a) *to upregulate the neurotrophin receptor *Ntrk2 *for survival via the neurotrophin *Bdnf *released from their target, the hair cells [108]. Among peripheral neurons, ear neurons of mice can be uniquely identified by the sequential expression of *Neurog1, NeuroD1, Pou4f1 *and *Ntrk2 *in combination with other factors such as *Gata3 *[109].

### More genomics/proteomics aspects of inner ear morphology

To unravel the true magic of the symphony of inner ear developmental control genes, we have to "listen" carefully to messages left by single and combined loss of function mutants of all the players. As indicated before, the inner ear is a very complex 3 dimensional structure, and patterning events resulting in that are orchestrated along three axes. To do so, the players must perform as a team. However, it appears that several major subgroups can be distinguished, with unique and overlapping patterning functions: (1) ***Hmx genes ***appear to act largely independently in their aspect of vestibular patterning (Figure 3) [13]. (2) ***The Shh-Pax *connection **induces and/or maintains ventral fate during the otocyst stage [84,110]. Mice deficient for *Shh *fail to develop cochlear structures. Recent detailed analysis of the inner ear defects in *Shh *null mice provides convincing evidence that Shh protein, secreted from the notochord, and *Pax2 *expression are crucial for specifying cochlear development following formation of the otic vesicle [68,84]. In the *Shh *null mice, failure of cochlear development is partly attributed to loss of *Otx1 *and *Otx2 *homeobox gene expression and primarily to lost otic epithelial expression of *Pax2*. Furthermore, the maintenance of *Pax2 *expression, by Shh protein, was shown to be critical in restricting the expression of the *Dlx5 *homeobox gene to the dorsal otic epithelium during cochlear specification. Interestingly, *Pax8 *expression was maintained in the otic epithelium of the *Shh *null mice, which strongly suggests that *Pax2 *and *Pax8 *are functionally independent of each other with regards to inner ear morphogenesis. Morphogenesis of ventrally and dorsally derived inner ear structures appears to degenerate due to decreased cell proliferation and simultaneously increased apoptotic cell death within the otic epithelium. If fact, the inner ear defects in the *Six1 *null mice are essentially phenocopies of those observed in both the *Shh *and *Pax2 *null mice, which includes lost *Otx1/Otx2 *expression and ventral expansion of *Dlx5 *and *Hmx3 *homeobox gene expression. Though at the molecular level, *Six1 *appears to be independent of the *Shh-Pax2 *pathway as their expression is maintained in the *Six1 *null mice and *Six1 *expression is maintained in the otic vesicles of *Shh *null mice. However, this does not rule out a disruption in *Shh-Pax2 *signaling via crucial protein-protein interactions.

**Figure 4:**
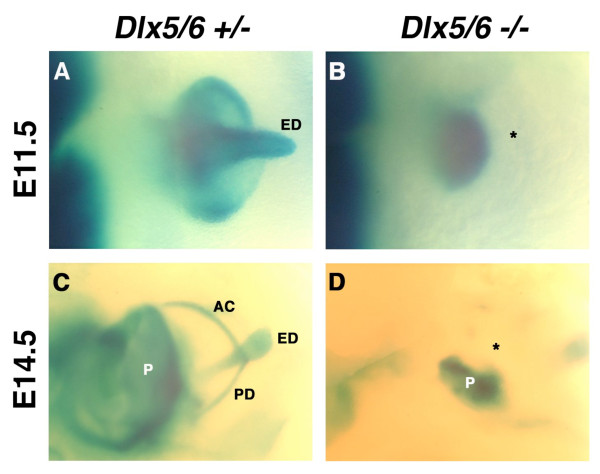
**Abnormal vestibular morphogenesis in whole-mount β-galactosidase stained E11.5 and E14.5 embryos lacking both *Dlx5 *and *Dlx6***. Embryonic *Dlx5 *and *Dlx6 *expression in the inner ears of heterozygous (A, C) and homozygous (B, D) *Dlx5/Dlx6 *embryos demonstrates that vestibular morphogenesis is arrested by E11.5 with the absence of presumptive semicircular ducts and endolymphatic duct (ED, asterisk). At E14.5, *Dlx5/Dlx6 *expression normally defines the majority of the vestibular apparatus, including the anterior (AD), posterior (PD), and lateral (LD) semicircular ducts, ampullae (A) and ED. In contrast, the cell lineage of the presumptive vestibular apparatus is absent (asterisk) from *Dlx5/Dlx6 *null embryos, which develop a rudimentary pinna (P).

Interestingly, an additional function of *Shh *seems the restriction of *Wnt *signaling to the dorsal otocyst [38]. Regulative interactions between *Shh *and *Wnt *signaling has previously been observed in other anlagen such as the neural tube [111,112], somite [113] and the limb [114,115] (3) **Wnt signaling **impacts on dorsal cell fate specification via *Dlx5/Dlx6 *and *Gbx2 *promotion, and indirectly ventral cell fate by restricting *Otx2 *ventrally via *Gbx2 *[77,80]. While recent studies by Riccomagno *et.al *describe the multiple roles of *Wnt *signaling during inner ear development [38], we decided to focus here on the important downstream targets *Dlx5/Dlx6 *in dorsal cell fate specification:

Thorough characterization of the previously reported inner ear defects in the *Dlx5/Dlx6 *double-null mice indicates that *Dlx5 *and *Dlx6 *have redundant functions during inner ear development [26,60]. More significantly, they are indispensable for the specification and morphogenesis of all vestibular structures (Figure 4). While otic induction proceeds normally in *Dlx5/Dlx6 *double-null embryos and an abnormal ventrally derived cochlea develops, morphogenesis of all dorsally derived structures that comprise the vestibule fail to arise in a manner that is in strong contrast to the *Dlx5 *null mice. Following establishment of the otic vesicle, the dorsal protrusion of the presumptive endolymphatic duct never forms and subsequent development appears to degenerate to the point that *Dlx5/Dlx6 *expressing cells become absent. Surprisingly, *Dlx5/Dlx6 *expressing cells are present in the presumptive cartilaginous otic capsules that surround a highly abnormal developing cochlea devoid of *Dlx5/Dlx6 *expression. The developing cochlea is completely encapsulated, highly dysmorphic, and appears to contain morphological sensory epithelia. Whether or not the sensory-like epithelium has any degree of functionality is presently unknown. As in the *Dlx5 *null mice, the vestibular defects seem to evolve from deficiencies in otic epithelial cell proliferation and increases in apoptotic cell death within the otic epithelium and the periotic mesenchyme.

The apparent failure of vestibular morphogenesis in *Dlx5/Dlx6 *double-null mice implies that multiple patterning mechanisms are defective in the otic epithelium and/or interactions with the otic epithelium are displaced. Unexpectedly, *Hmx2 *and/or *Hmx3 *expression in the otic epithelium are essentially unaffected prior to the onset of the severe morphological abnormalities observed in the *Dlx5 *null and *Dlx5/Dlx6 *double-null mice. Furthermore, no significant changes occur in the expression patterns of *Fgfs *or the genes that encode their receptors. However, expression of *Gbx2*, is completely absent from the otic epithelium prior to any morphological change. Suggesting that *Dlx5/Dlx6 *directly regulate *Gbx2 *expression in a manner that contributes to proper formation of the vestibular apparatus.

Expression of the *Msx1/Msx2 *homeobox genes is severely reduced or absent from both the otic epithelium and surrounding periotic mesenchyme of *Dlx5/Dlx6 *double-null mice. In contrast, the periotic expression domain of *Prx2 *is expanded which could indicate a compensatory consequence of lost *Dlx5/Dlx6 *function, since *Prx1/2 *double-null mice also have vestibular defects. Given that *Dlx5 *and *Dlx6 *are not normally expressed in the periotic mesenchyme, this suggests that they may regulate the activity of a secreted molecule that mediates epithelial-mesenchymal interactions during early otic morphogenesis. A likely candidate is *Bmp4 *since its expression is undetectable in the otic epithelium prior to any disruptions in the morphology of the developing otic vesicle. *Bmp4 *expression in the otic epithelium is thought to mark the development of the presumptive vestibular sensory cristae of the semicircular ducts. *Bmp4 *is normally expressed in two distinct patch-like domains in the anterior-lateral and posterior otic epithelium, which give rise to all three cristae of the three semicircular ducts [116]. Experimental use of the *Bmp4 *antagonist *noggin*, in chick, has indirectly demonstrated that *Bmp4 *plays a crucial role in sensory and non-sensory vestibular development, as all three semicircular ducts and their cristae are absent in the severe phenotypes [101,117]. In addition, Bmp4 protein is localized within the otic epithelium and secreted into the periotic mesenchyme during the morphogenesis of the mouse inner ear with the ability to induce and regulate otic capsule chondrogenesis [118]. This strongly suggests that *Bmp4 *is a critical mediator of epithelial-mesenchymal interactions that govern aspects of inner ear development. Furthermore, *Bmp4 *expression and subsequent secretion of its protein into the periotic mesenchyme is moderately to severely down regulated in the otic epithelium of the *Dlx5 *null mice. Therefore, *Dlx5/Dlx6 *may be specifying vestibular fate by regulating *Bmp4*, which has autocrine- and paracrine-like functions to promote sensory and non-sensory vestibular morphogenesis and to couple epithelial-mesenchymal interactions that initiate vestibular otic capsule chondrogenesis, respectively. *Six1 *may be an important modulator as its expression is required to maintain *Bmp4 *expression in the otic vesicle [74]. Additionally, *Bmp4 *could be orchestrating both local sensory and non-sensory vestibular morphogenesis and periotic mesenchymal initiation of otic capsule formation through direct regulation of *Msx1/Msx2 *expression, as in other organ systems [119-124].

An unforeseen consequence of lost *Dlx5/Dlx6 *expression is the complete otic epithelial expansion of *Pax2 *expression (Figure 2A). This is an exciting observation since Shh restricts the *Dlx5 *expression domain while activating *Pax2 *expression, which has no reported involvement in vestibular specification. In addition, the *Otx1 *expression domain is severely reduced to a small area along the lateral otic epithelium. Since the vestibular apparatus is absent in the *Dlx5/Dlx6 *double-null mice, it is likely that the remaining *Otx1 *expressing cells, along with *Pax2 *expressing cells, contribute to cochlea specification. It remains to be determined if *Dlx5/Dlx6 *directly regulate *Otx1 *or if its reduced expression domain is a secondary consequence. Since the onset of *Dlx5/Dlx6 *expression is later than *Pax2 *and prior to otic vesicle formation, *Dlx5/Dlx6 *may be critical regulators of otic cell fates by establishing compartmental boundaries through a means of region specific gene repression. Recent studies have determined that *Shh *and *Pax2 *are responsible for specifying cochlear development following formation of the otic vesicle in a manner that is complimentary to the vestibular actions of *Dlx5/Dlx6 *[68,84]. Wherein, the failure of cochlea development in *Shh *null mice can be attributed to lost *Otx1/Otx2 *and *Pax2 *expression in the epithelium of the otic vesicle. Interestingly, *Dlx5 *expression expands ventrally to include nearly the entire otic epithelium and *Bmp4 *expression is shifted in the presence of abnormal vestibular development. Thus providing experimental evidence that *Dlx5/Dlx6 *and *Pax2 *functionally antagonize one another through restricted otic epithelial expression domains that are critical in specifying vestibular and cochlear fates, respectively (Figure 2A and 2B).

To make an already complex signaling scenario even more complicated: Patterning of the inner ear does not rely on signaling events within the inner ear alone, but also depends on signaling clues from the adjacent hindbrain, neural tube and notochord [38,80,125], where sources for *Shh, Wnt *and *Gbx2 *can be found. This does not come as too big a surprise, given that the hindbrain is known to have otic induction capability and many hindbrain mutants also display inner ear defects [25,49,51,80].

Some recent studies have also attempted to look at global gene expression profiles of not only different structures such as the cochlea, utricle, and saccule within the inner ear but also at the temporal changes in these expression pattern[126]. Though these studies are useful in generating a gene universe, consisting of all the major genes and gene families involved in the development of an organ, it will require further analytical studies like chromatin immunoprecipitation to tie up these gene expression data with the interaction of the individual genes at different time points in development and will provide a clearer picture of the genomic events orchestrating the inner ear development.

### Some more recent players joining the group

In recent years there has been an increasing focus on non-coding RNAs and their role in development. Microarray analysis of microRNA (miRNA) expression in the postnatal mouse inner ear has revealed that at least 100 or approximately one-fourth of currently known mouse miRNAs are present [127]. Expression profiles are not substantially changed from the newborn mouse inner ear, through maturation and functional onset of hearing, and into adulthood, suggesting that miRNA expression is largely established in embryonic development rather than in later stages. Nevertheless, the abundance of miRNAs implies a considerable contribution to the regulation of genetic programs amongst the various cell types that are important to inner ear development, maturation, and function. A recent study used a *Pou4f3 *conditional Dicer knockout in mice, with a disruption in production and maturation of miRNAs specifically in the hair cells (HC) of the cochlea [128]. The severity of the HC phenotype varied along the cochlea, with HCs in the base showing more severe defects than those at the apex. In P38 mice many HCs lost their stereocilia (crucial for mechanotransduction), the apical surfaces of the residual inner and outer HCs became uniformly rounded and visibly reduced. In some other cases the HCs showed disorganized bundles of thin microvilli like stereocilia of uniform length and in some cases adjacent stereocilia fused together. By combining transcriptome profiling, in situ hybridization and bioinformatics the authors zoomed in on six miRNAs (*miR-15a, miR-18a, miR-30b, miR-99a, miR-182, and miR-199a*) showing different spatio-temporal expression in new born mouse cochlea and vestibule. Interestingly two of these miRNAs (*miR-15a-1 *and *miR-18a*) were also shown to be important in zebrafish inner ear development. By using bioinformatics tool the authors also identified *Slc12a2*, *Cldn12 *and *Bdnf *as potential targets for *miR-15a*.

## Conclusion

The formation of compartment boundaries has been proven vital in many anlagen and organisms during development and a 'compartment boundary' model was proposed for the inner ear [61]. Homeobox genes have shown diverse and widespread roles in the development of numerous organ systems. Existing and recent studies in mouse indicated that they are absolutely necessary for morphogenesis of the mammalian inner ear following otic placode induction. In craniofacial [65] and possibly limb development [25,26] the homeobox genes *Dlx5/Dlx6*, might exert a substantial role in boundary stabilization, aiding to restrict cells to a developmental compartment, an important function they could also engage in during inner ear development.

*Shh *specifies the ventral most cells of the otic epithelium that express *Otx1/Otx2 *and possibly *Pax2 *and which contribute to the morphogenesis of the cochlea. *Shh *also functions to maintain *Pax2 *and restrict *Dlx5/Dlx6 *to the medial wall of the otic vesicle, thus specifying cochlear fate. In addition, *Shh *and possibly *Fgf2 *function together in the periotic mesenchyme to initiate ventral otic capsule chondrogenesis via *Brn4 *and *Tbx1 *function [84,110]. *Dlx5/Dlx6 *specify the medial to dorsal most cells of the otic epithelium that give rise to the endolymphatic duct and vestibular apparatus (Figure 4). Vestibular morphogenesis requires the activation of *Gbx2 *and *Bmp4 *and may involve partial expression of *Otx1*. *Dlx5/Dlx6 *also function to restrict *Pax2 *expression to the medial wall of the otic vesicle epithelium. In addition, *Dlx5/Dlx6 *initiate interactions with the periotic mesenchyme via Bmp4 secretion from the otic epithelium, which then interacts with *Msx1/Msx2 *to provide positional control of the otic vesicle and dorsal otic capsule chondrogenesis. In essence, *Dlx5/Dlx6 *and *Shh *appear to functionally antagonize one another, through repression, to generate compartments of activities that specify distinct otic cell fates during the morphogenesis of the mammalian inner ear.

It is clear from existing data that homeobox-containing transcription factors, in conjunction with secretable factors, have wide ranging and critical regulatory roles for specifying the mammalian inner ear complex. Future studies are required to firmly establish the relationship between the *Dlx5/Dlx6 *and *Pax2 *genes for specifying the vestibular and auditory components, respectively, following otic placode induction and formation of the otic vesicle. At present, it appears that they functionally antagonize each other by defining their boundaries of expression within the three-dimensional otic vesicle, yet the mechanism of antagonism remains to be determined. In addition to activating expression of *Pax2 *(Figure 2A and 2B) does Shh, via secretion from the notochord, also act as a direct repressor of *Dlx5/Dlx6 *expression? What factors are acting upstream of *Dlx5/Dlx6 *during otic induction and upstream of *Shh *in the notochord? Likewise, does the secretion of Bmp4 within and out of the otic epithelium have any direct or indirect roles in specification of the auditory component of the inner ear? Additional experiments are required to substantiate the proposed interactions between *Dlx5/Dlx6 *and *Pax2 *homeobox genes and the secreted Shh and Bmp4 proteins in specifying the vestibular and auditory components of the mammalian inner ear.

It remains to clarify the functional roles of the *Hmx1/Hmx2/Hmx3 *homeobox genes, especially their role in the current model of inner ear specification. Since their expression domains overlap throughout the entire inner ear labyrinth and they are expressed prior to otic vesicle formation, the compartment model of inner ear specification predicts that they should be global regulators of inner ear specification. However, loss of function mutation studies in mouse has only revealed variable roles in vestibular development (Figure 3). In addition, their expression appears to be unaffected at the early otic vesicle stages in the *Dlx5/Dlx6 *null mice. Why is that and what is their position in the genetic hierarchy during inner ear specification? Are they upstream regulators and/modulators of *Dlx5/Dlx6 *and/or *Pax2 *expression during otic vesicle formation or do they form a redundant parallel pathway in vestibular morphogenesis? And what are their upstream regulators? In the near future these questions can be addressed by combining existing loss-of-function and conditional mouse mutant lines relevant to the developing inner ear. This will hopefully provide insight into the complex morphogenetic mechanisms that occur following otic vesicle formation and prior to cell fate determination in the developing inner ear. Though these are yet early days, there has been growing evidence supporting a resounding role for miRNAs in the developing inner ear. More studies will only lengthen the list of miRNAs expressed in the inner ear as well as their potential target at different stages of its development. By combining knowledge gleaned from the traditional knockout studies with recent studies focusing on the non coding RNAs and other regulatory sequences, we hope in the near future we will be able to 'listen to' and understand the complete symphony of inner ear developmental control genes.

## Authors' contributions

SC, PMK and TL prepared the figures and wrote the manuscript. All authors read and approved the final manuscript
